# “I need to terminate this pregnancy even if it will take my life”: a qualitative study of the effect of being denied legal abortion on women’s lives in Nepal

**DOI:** 10.1186/s12905-015-0241-y

**Published:** 2015-10-14

**Authors:** Mahesh Puri, Divya Vohra, Caitlin Gerdts, Diana Greene Foster

**Affiliations:** Center for Research on Environment Health and Population Activities (CREHPA), Kathmandu, Nepal; Advancing New Standards in Reproductive Health, University of California, San Francisco 1330 Broadway, Oakland, CA 94612 USA

**Keywords:** Denied, Legal abortion, Barriers, Nepal

## Abstract

**Background:**

Although abortion was legalized in Nepal in 2002, many women are not able to obtain legal services. Using qualitative data from women who were denied legal abortion services, we examined reasons for seeking an abortion, options considered and pursued after being denied an abortion, reasons for delaying seeking care, as well as complications experienced among women who were denied legal abortion.

**Methods:**

After obtaining authorization from two health facilities in Nepal, we requested informed consent from all women who were seeking abortion services to complete a case report form to determine their eligibility for the study. We then recruited all eligible and interested women in to the study. Two months after recruitment, we conducted in-depth interviews with 25 women who were denied abortion services from the two recruitment facilities due to advanced gestational age (>12 weeks). Interviews were translated and transcribed, and the transcripts were analyzed through an iterative process grounded in thematic analysis, involving both *a priori* and emergent codes.

**Results:**

Eleven women were recruited from the government hospital and 14 from an NGO facility. The majority of women (15 women or 60 %) were living rural settings, ranged in age from 18 to 40 years and had an average of 2 children. None had completed any post-secondary education. Women most commonly cited financial concerns and health concerns as reasons for seeking termination. Not recognizing pregnancy, uncertainty about how to proceed, needing time to coordinate the trip to the facility or raise money, and waiting to know the sex of fetus were the commonly cited delays. Among the women interviewed, 12 decided to continue their pregnancies following denial, 12 terminated their pregnancies elsewhere, and one self-induced using medication. At least two women experienced significant complications after obtaining an abortion. Most women who continued their pregnancies anticipated negative consequences for their health, family relationships, and wellbeing.

**Conclusions:**

Barriers to seeking early abortion need to be addressed in order to reduce utilization of abortion services that may be unsafe and to improve women's health and wellbeing in Nepal.

## Background

Access to safe abortion services and to post-abortion care are critical to women’s ability to control their fertility, protect their health, and ensure the wellbeing of their families [1–3]. In many countries, legal abortion is available within certain gestational limits [4], and women who seek care beyond the legal limit are turned away [5, 6]. Researchers have hypothesized that, in such settings, women who are denied abortions may go on to seek illegal abortions elsewhere [5–8]. However, due to a lack of evidence on the experiences of women denied abortion services outside of the United States, it is unclear whether women seek alternative services after denial. Furthermore, the health consequences of seeking alternative, and potentially unsafe, services are undocumented [8].

Abortion was legalized in Nepal in 2002, and abortion services were established at almost all government hospitals, designated private hospitals and non-governmental organization (NGO) clinics following the passage of the Safe Abortion Policy in 2004. The law allows medical and surgical abortion up to 12 weeks gestation on request, up to 18 weeks if the pregnancy results from rape or incest, and at any time during pregnancy if the physical health, mental health, or life of the woman is at risk, or the fetus is impaired/has a condition incompatible with life [9]. Previous laws did not allow abortion under any circumstances and many women were imprisoned for having abortions [10]. National efforts to scale-up abortion service provision in Nepal have enabled nearly 500,000 women to obtain safe, legal services between 2004 and 2011 [10]. Over the past ten years, the Nepali government has taken important steps to include abortion as a component of women’s reproductive health services; nevertheless, access to abortion services continues to be challenging for many Nepali women, especially the poorest, marginalized, and geographically isolated [11].

The reasons why Nepali women seek abortion can vary greatly, and are known to include completion of desire family size and mistiming of pregnancy, lack of knowledge about where to obtain reproductive health services, concerns about finances, and in some cases, concerns about the sex of the fetus [12–14]. The majority of Nepali women have little or no knowledge of the abortion law and many do not know where to obtain safe abortion services [15, 16]. In addition to lack of knowledge about the law, Nepali women also face challenges in accessing legal abortion services. Abortion facilities, including those in this study, are mostly concentrated in urban areas and at district headquarters. Despite efforts to increase the availability of and access to abortion services in facility catchment areas [17], it is often difficult for women living in rural areas (the large majority of women in the country and 60 % of the study population) to travel to clinics [10].

Additionally, abortion services come with a cost, even at government facilities. Despite the 2009 Supreme Court’s Order to ensure that all Nepali women have access to safe abortion services, the government has not created an effective mechanism through which to provide cost-free access to abortion services for poor and marginalized women, and fees are often prohibitively expensive [10]. The government has recently announced that abortion services will be made available free of cost in the public facilities, but this will take time to implement. Although there is sufficient evidence that mid-level providers such as nurses and midwives can provide medical abortion as safely and effectively as physicians, the government has also been slow to scale-up training of such providers, a move which could greatly expand the numbers and locations of abortion providers [11, 15–18]. Finally, while complications from unsafe abortion have declined over the past five years in Nepal, complications due to the use of unknown or unsafe medications, often dispensed from uncertified sources, remain a major concern [19, 20].

In this paper we present data from a qualitative study conducted among women who were denied legal abortion services in Nepal. The study sought to examine reasons for denial of legal abortion, options considered after denial, sources of information about illegal abortion, experiences seeking illegal abortion, abortion complications experienced, and post-abortion care seeking.

## Methods

We conducted in-depth interviews with 25 women who were denied abortion services due to higher gestational age (more than 12 weeks) from two health facilities in Nepal, in September and October of 2013. The study sites – a reproductive health non-governmental organization (NGO) clinic located in a sub-metropolitan region in Eastern Nepal and a major tertiary government hospital in Kathmandu – were selected to represent different types of health facilities that serve substantial numbers of abortion and post-abortion patients in diverse geographic settings. Participants were eligible for the study if they were women between 18 and 49 years old, seeking abortion services, and denied services due to advanced gestational age on the day of recruitment. Women were further selected for interviews based on their geographic proximity to the recruitment sites (living not more than 48 h from the clinic), in order to increase the likelihood of a successful follow up in person interview two months after recruitment.

After routine intake, counseling, education, screening and ultrasound procedures (if needed) at the recruitment facility, the clinic staff informed potentially eligible women about the study and pointed her to study staff. The study staff briefly described the study to potential participants and handed out flyers with basic information. Study staff then obtained informed consent from those interested in participating and asked them to fill out a case report form containing basic socio-demographic information. A provider at the health facility also noted the reason for denial of abortion services on the case report form. Women who fulfilled eligibility criteria were invited to participate and informed that participation would include a voluntary, confidential interview two months after recruitment at a preferred place and time. For those who opted to enroll in the study, study staff collected detail contact information and used this information to contact participants to schedule an interview after the initial clinic visit.

Interviewers trained in qualitative methods for abortion-related research traveled to participants’ homes, or another location of their preference, where they conducted the interview in person in Nepali. Participants were informed that all data would be de-identified prior to coding, dissemination and publication and that they had the option to decline an interview at any time. Study staff obtained written consent or thumb print (for women who could not sign their name) from all participants. Participants were provided a small gift, valued at two U.S. dollars, for their participation in the study. Ethical approval was obtained from the Nepal Health Research Council in Kathmandu, Nepal. The University of California, San Francisco Committee on Human Research issued a certificate indicating that all data analyzed by UCSF investigators were de-identified and the analysis did not require further IRB review.

In-depth interview guidelines were developed in English and translated into Nepali. The interview guide was open-ended, which enabled the interviewer to adapt it to the particular participant during the interview. Revisions to the interview guide were made as needed based on the first interviews. Topics included decision-making processes around unintended pregnancy and abortion, experiences with abortion seeking and abortion denial, subsequent attempts to obtain abortion (if any), impact of denial on future plans, family, and wellbeing, knowledge about the abortion law, and advice for others seeking abortion.

Interviews were digitally recorded, transcribed verbatim and translated from Nepali to English. A thematic analysis approach was used, whereby key themes in the data were identified, coded, and analyzed. Texts were coded using an iterative process, with U.S. and Nepali research team members discussing and evaluating the codes and meaning of the data. *A priori* codes were first established based on the interview guide. Emergent codes were then developed based on themes and topics that arose from the data after several readings of the transcripts and interviewer memos. Axial codes were then used to connect codes to one another. Final coding was primarily conducted by one US-based co-author trained in qualitative analysis, with an additional US-based researcher coding a subset of seven transcripts to ensure the reliability of the coding process and clarity of the codebook. Finally, findings were summarized across themes. For the purposes of identification in the quotes presented below, respondents who were continuing their pregnancies are labeled with a “C” (followed by their identification number) and respondents whose pregnancies were terminated are labeled with a “T (followed by their identification number)” The computer software program Dedoose 4.5 (Los Angeles, CA) was used to analyze the transcripts.

## Results

### Profile of study participants and pregnancy outcomes

**Fig. 1:**
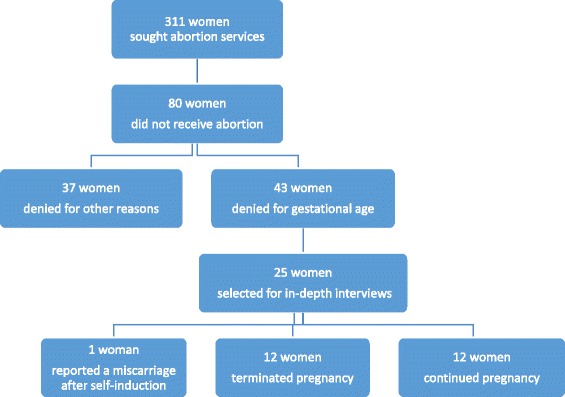
Recruitment and Participant Selection

A total of 311 women (149 from the government hospital and 162 from the NGO clinic) sought abortion services from the two sampled facilities in September and October 2013 (Fig. 1). Of these, 80 women or 26 % (51 from government hospital and 29 from NGO clinic) did not receive the abortions they were seeking; 43 (24 from government hospital and 19 from NGO clinic) were turned away for advanced gestational age (14 % of the women seeking services) and 37 (27 from government hospital and 10 NGO clinic) for other reasons such medical contraindications, small gestation size (less than 6 weeks) and ultrasonography not available at the time of service (12 % of the women seeking services). The characteristics of these women have been described elsewhere [21]. Of the 43 women who were denied for advanced gestational age, 25 women were selected purposively based in part on their place of residence, caste and ethnicity and level of education to have diversity in the sample for participation.

All 25 women who were recruited for in-depth interviews consented to participate. Of this sample, 11 women (44 %) were recruited from the government hospital and 14 women (56 %) from the NGO facility. The majority (15 women or 60 %) were living in rural settings and 10 women (40 %) were living in urban settings.

The women ranged in age from 18 to 40 years and had an average of two children (with a range from 0 to 6; three women were nulliparous). None had completed any post-secondary education. Eight women were illiterate or reported only knowing how to write their own name. The majority of women (13) were housewives; others worked in business, agriculture, or daily-wage labor.

Of the 25 women interviewed, 12 were continuing with their pregnancy. Of the remaining 13 women, 12 terminated their pregnancies elsewhere after being denied legal services at a recruitment site and one woman reported that she experienced a miscarriage one week after attempting to self-induce with medication.

### Reasons for considering abortion were varied

Women gave multiple reasons for seeking an abortion, including financial constraints, the need to look after other children, maternal age, health concerns, and sex of the fetus. There were no apparent differences in the frequency or importance with which these concerns were cited by women who did and did not eventually terminate their pregnancies. Some women emphasized the desperate nature of their situations as they discussed their reasons for wanting an abortion. For example, one woman gave several reasons for wanting to end the pregnancy (she had adult children and thus felt she was too old to have a baby, and she was very concerned about her family’s precarious financial situation). She said:

*“I did not have money to go to the doctor for abortion. How was it possible without money? I was unable to go for a check-up as I had taken loan and I could not use that money. Later I was really tense thinking about how I would do it. I was in a state where it was impossible for me to live*.”(T15)

Sex selection (specifically a preference for a male child) was cited by four women as a primary reason for seeking an abortion. Of those four participants, one woman (C18), sought to terminate her pregnancy elsewhere after being turned away from a recruitment site, but then decided to continue her pregnancy when, at her second appointment to terminate her pregnancy, she learned she was having a son:

*I: What did your husband say when they said it was a son after the check-up?*

*R: My husband was happy, we have three daughters and we needed a son. Everyone would have dominated us if we did not have a son. That is why my husband is very happy.* (C18)

The other three participants (C01, T12, and T25) wanted to terminate their pregnancies once they had an ultrasound and learned they were having girls, but were told that at that point, they had exceeded the legal gestational age limit; two were subsequently successful in obtaining abortions at private facilities (T12 and T25). A participant summarized why chose to terminate based on the sex of the fetus:

*“My mother in law…used to tell me that my brother in law had two sons. A son is a son. What is the point of having a daughter because we love them for two days and after marriage they will go to another house… I already had two daughters and I do not want another. If I gave birth to another daughter then I would have three daughters and having three daughters would be hard for us. What if we can’t raise her and educate her properly? That’s why we went for an abortion as everybody else was doing it”.* (T12)

### Pathways to abortion decision-making are complicated

The majority of women were clear about how they felt about the pregnancy at the time they were interviewed, but the process of arriving at their decision to terminate could often be complicated. Some originally considered continuing the pregnancy, but later changed their minds. Most women reported weighing many competing factors when making their decisions, including concerns for the health and well-being of existing children, financial constraints, expectations from family members, their current gestational age, and the desire to carry a pregnancy to term.

*“I felt like I should keep it when other people started to shout at me saying it would be a sin to [abort]. After that I kept thinking about it and my child lost weight because she was unable to drink my milk and then I thought […] it would be difficult to educate them. We have to educate daughters more than sons because I had to face so many hardships as I was not educated. So I thought I would not be able to educate them and I also felt like, what we would do with three children?”* (C05)

*“But somewhere in my heart I wanted to deliver that baby and I wanted to have a daughter also. Whenever I see others; daughters even I wish to have a daughter. My husband has three siblings but all of them don’t have daughter so I had wished that baby was a girl. But later on I started to feel like I am old and [giving birth] was life threatening too ….” (*T07)

Seven women explicitly reported that they had difficulty making the decision to terminate the pregnancy. For example, a woman said:

*“It was really difficult to make that decision. I sat in [the temple] and thought for a long time. I thought like if I gave birth to that baby then other kids would face problems for their whole life. I have brought up my kids and they are studying. I have been experiencing all that. Even that wish to have a new kid would make my children doubt me. They ask me why I needed three kids. For that I tell them, that is my wish. But in terms of that baby, how could I deliver that kid as I already have sons and daughter! We have to think about others as well. Only one’s desire doesn’t work”.* (T06)

Similarly, another participant felt differently about having an abortion when she learned how far along she was in her pregnancy:

*“When they told us that the baby has already developed and grown I had a different feeling. I felt like it has already grown and in second thoughts I also felt that if it is possible to abort then I should. But when they said it was not possible I was okay with it”.* (C02)

Although many participants reported receiving advice from a variety of sources (health providers, family members, friends), the decision-making process was often strongly influenced by women’s husbands. Several women indicated that the decision was actually up to her husband, and was not her own, reflecting the reduced reproductive decision-making that many women face in this context [14]. For example, two participants reported, “I will have to do whatever my husband says,” (C18), and, “It was [my husband’s] decision, not mine” (C19). The other common source of advice came from sisters, sisters-in-law, cousins, neighbors, friends, or even nurses or social workers. As a broad category, “sisters” could be very influential. Like husbands, “sisters” tended to have many strong opinions about what a woman should do about her pregnancy. Unlike husbands, these women were often also a source of practical information about where a woman could obtain an abortion, what methods could be used, and the perceived safety and efficacy of certain methods. For example:

*I: Who suggested you to go to … Hospital?*

*R: I talked about that with my sister, the one with whom I had talked over phone earlier […] She is my third sister. She told me that she had visited that hospital when she hadn’t been able to deliver her baby. She said that it was easier there.* (C09)

*I: before making a decision regarding abortion did you talk to anyone?*

*R: No…. While I was returning back from school I asked a social worker, there is an aunt who is a social worker so I went inside and asked her what would happen if I did such things and she said if I did it through good doctors then it would be good. That was the only suggestion that I got.*

*I: What else did the social worker say?*

*R: She said if I wanted to do it then I should go to a good doctor and do it safely. If I take medicines that are available in the shops then something could happen in the future.*

*Then I asked her, won’t it be helpful if I go to a good doctor? And she said that I should not be very scared, just a little.* (T20)

Many reported telling only their husbands about the pregnancy, though at least half of all of the women interviewed had also talked to other close family members. A few women said that they were worried to tell their husbands. Two women in particular (T16 and T25), lied to their husbands and told them they had miscarried. For example:

*“I did it [abortion] with my own wish and my husband doesn’t even know about it. I have told my husband that I had a miscarriage and he believes me. After that he has never asked me about it”.* (T16)

Similarly, many women said that they had never heard of anyone else having an abortion, an issue that might explain their own reluctance to talk to many people about their abortion. For example, a woman who got an abortion had this to say about her community:

*I: How many women you know might have had an abortion?*

*R: Who would talk about such problems? No, in our village they go to the doctor for such problems and when the doctor says it cannot be done they come back home and give birth to the baby.* (T13)

One woman specifically sought an abortion at a health clinic because she was concerned that taking medicines at home would reveal that she had had an abortion:

*“I just had thought of visiting private centers and taking the medicine to end my pregnancy. But then I realized that such medicine would cause bleeding and if I bled a lot then others would know about the abortion. So I wanted to do that secretly*”. (T10)

### Pursuing abortion can take time

Women cited many reasons for delays in seeking abortion care: not realizing they were pregnant, uncertainty about how to proceed, needing time to coordinate the trip to the hospital and/or money to pay for services, and, for those who specifically wanted a son, waiting to know the sex before deciding about termination. An example of a delay due to financial constraints:

*“I didn’t have much money for abortion, without money it wasn’t possible for me. I didn’t have any source, so I worked for a whole month and collected money for abortion, then only it was possible…. I collected 2500 Rupees and I also had to arrange food for some time as labor work wouldn’t be possible after abortion. I needed two months of complete bed rest because of weakness.”* (T14)

Similarly, an explanation for delays in seeking services:

*“I had thought of going for the checkup but then sometimes I had to go to cut grass for the cattle and sometimes I had to graze them. In that way time simply flew away. On top of that the health center is not near here.* (T11)

Many women had already visited at least one other health facility before arriving at one of the two facilities where they were recruited for this study. Women most commonly went to private clinics or lower-level government facilities, and when those facilities couldn’t or wouldn’t provide the abortion, they were instructed to go either to the NGO clinic or to the regional level hospital. Some women visited multiple public and private health facilities before arriving at one of the two facilities where they were denied services again and recruited for this study.

Three women unsuccessfully tried to self-induce using medicines (T11, T21, and C24) before seeking abortions through the health system. All three seemed to have similar experiences: they took the medicines they were given and thought they had successfully ended their pregnancies, but then they missed a period, went to the doctor, and were told they were still pregnant. A woman explains:

*I: What had happened after you took the medicine?*

*R: I don’t remember, after taking the medicine I was bleeding for three days and then it stopped.*

*I: What happened after that, can you remember and tell us?*

*R: My husband was also sure that the pregnancy was terminated when it started bleeding; we thought it was over…*

*I: When did you know that you were still pregnant?*

*R: When menstruation stopped.* (C24)

One of these women (T11) went on to get an abortion at a private clinic. Another woman (C24) continued her pregnancy. The third woman reported that she and her husband decided to continue the pregnancy after learning that their medications were unsuccessful and that the fetus was apparently healthy. She later miscarried:

*“My husband also said that if the baby was fine we should give birth. He said whatever mistakes we made, now we should give birth, which is why we went for the check-up. One week after taking the medicines I started to have a stomachache. The next day we went to… hospital early in the morning and I just had a miscarriage”.* (T21)

Many other women knew about self-induction methods, such as obtaining medicines from pharmacies, massage (or “squeezing”) and use of “sticks or pipes”, though they did not attempt it themselves. One woman was interested in trying it but was unable to obtain the medicines:

*“I went to medical shop and asked how much it would cost for the medicine and they said it would cost 1500 rupees. They asked me how many months and when I told them they said it was not possible there as I had exceeded the pregnancy period. Even they were scared that they might get arrested. As I had exceeded so many months and the abortion could risk my life, they didn’t agree to give me the medicine”.* (T14)

Others who had heard that it was possible to self-induce said that they didn’t attempt it because it seemed dangerous or unlikely to work, or because they didn’t know what medicines they needed or where to get them.

*I: Your friends had told you that abortion can be done by taking medicines as well so didn’t you try those suggestions?*

*R: […] I doubted if abortion could be done by taking medicines. I had also heard from someone that it does not work.* (C01)

### Actions and emotions upon being turned away varied

Because each respondent’s case report form noted the provider’s reason for denying abortion services, the research team knew why respondents had been turned away. However, when women were asked if they knew why they did not receive services, their responses varied. Most women reported that they knew they had been turned away from abortion services due to advanced gestational age. Some were not entirely certain if this was the only reason they were turned away, but most thought that gestational age must have played a role. Despite probing, many women seemed to find it difficult to express their feelings upon being turned away. One woman (T07) was frustrated because she said that she was made to believe that the doctor would perform the abortion, but when she returned the next day with sufficient money to pay for the abortion, she was turned away:

*R: The doctor was ready to terminate my pregnancy but then I didn’t have money so I asked him if I could visit him on the next day. Then he said ‘ok’ so […] on the next day I went there with my brother-in-law but then he said that the baby couldn’t be aborted. I was disappointed then…*

*I: You are saying that you had your video x-ray done on the first day and got your report too.*

*R: Yes. And [the doctor] had agreed to abort the baby. But on the next day it seemed like pregnancy had crossed its time. On the next day, the doctor told me that they could terminate the pregnancy below 12 weeks. But on that day mine had crossed 12 weeks. Then I felt sad…”* (T07)

Some women were angry or upset upon denial of abortion services. One participant was told that performing an abortion at her current gestational age would be dangerous, which made her angry because she felt they were not accurately weighing this danger against the danger of giving birth:

*“I got really angry as the doctor had said all this and later what would become of me when I had to give birth. The only thing that I keep thinking of is, how I will give birth to the baby later?”* (C04)

Others felt scared and desperate when they were denied care. One woman explains:

*“I felt very scared when the doctor told me it wasn’t possible. When they said ‘no’, I felt like, where am I going to go and how would I survive in this state? I felt like crying and I cried too. I told her I needed to terminate this pregnancy even if it will take my life. I told her [Female Community Health Volunteer] that I was ready [for an abortion]”.* (T14)

Despite these emotions, many participants trusted the judgment of the doctors and accepted that they would not be receiving the services they wanted: For example:

*R: I just thought that they really couldn’t abort the baby so they had sent me back....if something is not possible then how can we force them to do that?* (T06)

*I: How did you feel when you did not get the services at the hospital?*

*R: I felt as if it was their wish to do it or not.* (C17)

Upon being denied abortion services, women typically received some advice from their providers. Health providers at the facilities where women were turned away were, in fact, often the ones to suggest private hospitals that would provide the abortion, although these referrals were often accompanied by judgment or sometimes misinformed warnings that the procedures, or that the act of obtaining an abortion in general, may not be safe. For example:

*I: What did they say?*

*R: They said if I [abort] now then some people also suffer from cancer because of it.*

*I: Why?*

*R: If one [aborts] too much then one can get cancer.*

*I: Had you done it before as well?*

*R: No, I have not; they just said that if one [aborts] too much then cancer can happen and they also said that I was too weak and yet if I wanted to [abort] then I could go to another place somewhere in [neighborhood].* (T05)

Providers who did not offer suggestions for where to obtain an abortion typically advised women to continue the pregnancy. Adoption was not discussed in any of the interviews.

Most women, who continued their pregnancies, said that carrying the pregnancy to term would not affect their relationships with other family members, or that they had not considered the possibility. A small minority thought that a new baby would cause conflict in their marriages. One woman said:

*“I told him [my husband] I will keep the child although he doesn’t want it. I also told him that I will live by myself even if he doesn’t look after me”.* (C03)

One woman thought that the new baby would improve her relationship with her in-laws, who had not wanted her to pursue an abortion:

*“Even they tell me to give birth and they tell us who will look after us and raise the children later. If you give birth then we are here and they tell me that will look after the child and support us”.* (C23)

When asked more specifically about any worries or concerns about raising a new child, women who were continuing their pregnancies often reported being worried about finances and resources:

*“Economically, raising two children is so hard and adding another is harder. Physically also it would affect me as I already delivered two children through caesarean. Again I have to deliver the third child by operation and it’s going to be more difficult. I am worried all the time about raising my small children”.* (C02)

*“It will highly affect me. For 3 or 4 years I will not get to go out as I will have to look after him/her. We don’t get food without working but I will have to be with the baby. I will have to live that way. What to do?”* (C09)

### Getting an abortion after being turned away is not always easy

Twelve out of 25 women (48 %) successfully obtained abortions at private facilities for a cost after being turned away from the recruitment facilities. The process of obtaining these abortions was often quite complicated. Many women received multiple referrals and visited several private health facilities before finding one willing to perform the procedure at an affordable price. It was often unclear to women whether or not the facility was authorized to provide abortion and they were not adequately informed about what to expect with regard to the protocol, procedure and standards of care.

Women commonly paid between 10,000 and 15,000 Nepali rupees (US$100 and US$150) for abortion services. Some women reported that the cost increased with gestational age. The most that anyone paid was 26,000 Nepali Rupees (T10, US$260) and the least was 2,500 Nepali Rupees (T14, US $25); in this case, a Female Community Health Volunteer negotiated a lower price on her behalf, informing the doctor that she was very poor. Women described receiving medicines and having surgical procedures, but most did not have a clear memory of the process and were not given much information from the health providers. One woman said: ‘They fed me medicine. The way they aborted the baby was… they placed [medicine]’ (T11). Another woman described in more detail:

*R: They did it by using their hands and a machine. There were two nurses. With the help of the machine the doctor finished my abortion. One of the nurses was always with me thinking I might get scared. […] I had a stomach pain at that time and she put hot water bag on my stomach and gently massaged over my stomach. They let me rest on the bed for 20 min. Then the doctor told me to walk slowly and leave. I bought vitamins worth150 rupees and I felt better. I also ate meat soup that made me stronger.* (T14)

### Many unaware about abortion law and legality of services

The vast majority of women who were interviewed reported that they had no knowledge of abortion-related laws in Nepal. Many knew that health facilities typically did not perform abortions after 12 weeks, but women did not necessarily recognize this as a legal restriction. For example, a woman said that neither she nor her husband had heard of the laws regarding abortion, but reported that the following information was given to her at the (hospital):

*“What they said was that to abort pregnancies that have exceeded 12 weeks the baby has to be weak while in the womb. Then only abortion can be done; otherwise it can’t be done”.* (C04)

Others were fairly certain that abortion is illegal in Nepal:

*“People say it is illegal so I am scared.”*(T11)

*“The police will arrest you if they know about it and in such a situation the nurse involved in abortion will also be arrested.”* (C24)

All of the women who obtained abortions after being turned away from the recruiting sites were beyond the legal gestational age limit for abortion on request, and none of the women mentioned any of the exceptions to the abortion law (rape, incest, physical and mental health or fetal anomalies) as reasons for abortion-seeking. Further, all of the women who obtained abortions did so at facilities that were unlikely to have been certified to legally provide abortion services. However, only one woman explicitly identified her abortion as illegal; she said:

*They disposed [of the fetus] and told me not to tell about it to anyone as it was illegal. They asked me who gave me their number and I told them a sister from [the town] had given me their number. They kept it undercover regardless of who came…(T13).*

As previously mentioned, women rarely knew whether abortions were ever legal, or under what circumstances they could be considered legal; some believed that seeking an abortion came with significant legal risks, in addition to social or medical risks. Yet, all of these women felt that their need for an abortion was worth these risks. For example, a woman (T06) describes obtaining an abortion even after learning that she was beyond the legal gestational limit and was warned of health risks:

*I: What did you hear?*

*R: Abortion is not possible after 12 weeks of pregnancy […] Even the clinic people said ‘no’ but we told them our problems and made them do that. They told us not to complain afterwards if anything went wrong.* (T06)

### Complications and quality of abortion care

Two women (T13 and T16) experienced significant complications after their abortions. One woman (T13) bled for 6 weeks after her abortion but did not want to return to the facility for additional treatment because of her negative experience there. Instead, she tried to treat herself:

*I: Did you go anywhere for the treatment of that problem?*

*R: I did not go anywhere; I just took the medicine available in the village and stayed. I bought the medicine from the grocery store in the village, [ibuprofen] I used to take two each every day. I took it for 6 days. I cannot do heavy work as my stomach pains. When I do household work or even when I work in the field I have to rest for a while.* (T13)

Another woman (ID16) bled for one month after her procedure, but she returned to the facility for additional treatment, which resolved the issue:

*I: What did you after going there?*

*R: They looked at it and said small parts had remained inside. Small bones and parts of it had remained inside which is why it was bleeding so they said they would clean it again and did it. 5–6 days after that everything was fine.* (T16)

Overall, women who received abortions typically reported being happy, relieved, and satisfied with the services they had received. When probed further, some women reported being treated poorly at the private clinics where they received their abortions. For example, one woman recounted:

*“It is difficult when a person is uneducated. Had we been educated and known how to speak they would have treated us nicely”.* (T13)

Another woman pointed out that women seeking antenatal care are treated very differently from those seeking abortions:

*“[The nurses] said we give birth to many children and then do many abortions. Women who gave birth were treated well because when I was in the other ward nobody said anything to me. I went there for operations twice and it was like staying at home with the nurses and doctors. […] but on the other side they shout at us. I did not like the abortion ward, they were a little rude. We don’t go for abortion purposely to risk our lives, it happens unknowingly. […] But the sisters on the other side treated me better.”* (T05)

## Discussion

Our findings represent the first effort to study the experiences of women who sought but did not receive legal abortion services in Nepal. Understanding the process of pursuing an abortion, being denied legal services, and making decisions about subsequent options will better inform the delivery of abortion services and the implementation of the law in Nepal. Half of all women in our sample obtained an abortion after being denied legal services. None of the women in our sample reported returning to the recruitment facility or pursuing the additional approvals necessary to obtain a legal abortion beyond the gestational age at which abortion is available on request in Nepal. This finding highlights the relative frequency with which abortions may be performed outside of the legal framework in Nepal. This finding also suggests that even women who are aware of the legal status of abortion in Nepal do not fully understand the scope of the law, and that the limits of the existing law, specifically the 12-week gestational limit, may prevent many women from receiving the abortion services they need. This is consistent with an earlier study which found that Nepali women’s knowledge of the abortion law was limited and that many had sought abortions from uncertified providers [20].

Many women face significant financial and logistical constraints, including referrals to multiple facilities. These delays prevent many women from receiving legal abortion services within the first 12 weeks of pregnancy when abortion is available on request in Nepal. After being denied a legal abortion, women in our study perceived that their only available option for pregnancy termination were abortions offered by private health providers who likely were not legally certified to perform the abortions they offered. In our study we found that the abortions that women obtained after being turned away at the recruitment facilities were associated with risk: at least two women in our sample experienced significant complications after their procedures. Unsafe abortion is an issue that continues to be of concern in Nepal [19]. This finding highlights the potential for such abortions to result in serious injury or death. Women also experienced fear of judgment or stigma from family members, community members, and health providers about pursuing abortion in general. The fact that women overcame these access barriers and poor quality of care illustrates how much they were willing to risk to obtain abortion services.

Overall, our findings point to the fact that access to high quality, legal abortion services may be limited in Nepal in spite of a relatively liberal abortion law. Many women reported having a limited understanding of their rights under this law, and some knew very little about the safety and availability of abortion services more generally. Furthermore, women faced significant barriers when seeking abortion services, including large financial and logistical burdens, concerns about the safety of the procedures they were receiving, and poor treatment by health providers in both certified and uncertified settings. The frequency with which such barriers were described by study respondents highlights the fact that more work must be done in order to provide safe, legal abortion services to all women who seek them.

While our data shed light on important issues related to abortion access in Nepal, our small sample size, purposive sampling strategy, and qualitative data limit the generalizability of our findings. Women were selected for interviews based in part on how difficult it would be to travel to their homes and the perceived likelihood that they would participate in an interview at the scheduled time, which may help to explain the study staff’s success with interviewing the women who were recruited. Women who lived further away or were otherwise harder to reach were likely to have had very different experiences seeking and obtaining abortion services, but these experiences are not well documented. Similarly, we were not able to examine the experiences of women who only sought unsafe or illegal abortions and never presented at one of the two legal abortion facilities which recruited for this study.

## Conclusions

This study provides new and important information about the experiences of women who seek abortion services in Nepal. Our findings point to the need for systematic, quantitative evidence on the risk factors for presenting later in pregnancy, predictors of seeking unsafe illegal abortion, and the health consequences of illegal abortion and childbirth after an unwanted pregnancy [18–21]. Such data could help to identify strategies to improve access to abortion services in Nepal and similar settings in which abortion is legal.
